# Cost-effectiveness of collaborative care for chronically ill patients with comorbid depressive disorder in the general hospital setting, a randomised controlled trial

**DOI:** 10.1186/1472-6963-7-28

**Published:** 2007-02-26

**Authors:** Eva K Horn, Tjeerd B van Benthem, Leona Hakkaart-van Roijen, Harm WJ van Marwijk, Aartjan TF Beekman, Frans F Rutten, Christina M van der Feltz-Cornelis

**Affiliations:** 1 Netherlands Institute for Mental Health and Addiction (Trimbos-institute), Utrecht, The Netherlands; 2 Onze Lieve Vrouwe Gasthuis, Amsterdam, The Netherlands; 3 Institute of Medical Technology Assessment, Erasmus Medical Centre, Rotterdam, The Netherlands; 4 Free University Institute for Extramural Research, Amsterdam, The Netherlands; 5 Department of Psychiatric Epidemiology and Department of Psychiatry, Free University, Amsterdam, The Netherlands

## Abstract

**Background:**

Depressive disorder is one of the most common disorders, and is highly prevalent in chronically ill patients. The presence of comorbid depression has a negative influence on quality of life, health care costs, self-care, morbidity, and mortality. Early diagnosis and well-organized treatment of depression has a positive influence on these aspects. Earlier research in the USA has reported good results with regard to the treatment of depression with a collaborative care approach and an antidepressant algorithm. In the UK 'Problem Solving Treatment' has proved to be feasible. However, in the general hospital setting this approach has not yet been evaluated.

**Methods/Design:**

CC: DIM (Collaborative Care: Depression Initiative in the Medical setting) is a two-armed randomised controlled trial with randomisation at patient level. The aim of the trial is to evaluate the treatment of depressive disorder in general hospitals in the Netherlands based on a collaborative care framework, including contracting, 'Problem Solving Treatment', antidepressant algorithm, and manual-guided self-help. 126 outpatients with diabetes mellitus, chronic obstructive pulmonary disease, or cardiovascular diseases will be randomised to either the intervention group or the control group. Patients will be included if they have been diagnosed with moderate to severe depression, based on the DSM-IV criteria in a two-step screening method. The intervention group will receive treatment based on the collaborative care approach; the control group will receive 'care as usual'. Baseline and follow-up measurements (after 3, 6, 9, and 12 months) will be performed by means of questionnaires. The primary outcome measure is severity of depressive symptoms, as measured with the PHQ-9. The secondary outcome measure is the cost-effectiveness of these treatments according to the TiC-P, the EuroQol and the SF-36.

**Discussion:**

Earlier research has indicated that depressive disorder is a chronic, mostly recurrent illness, which tends to cluster with physical comorbidity. Even though the treatment of depressive disorder based on the guidelines for depression is proven effective, these guidelines are often insufficiently adhered to. Collaborative care and 'Problem Solving Treatment' will be specifically tailored to patients with depressive disorders and evaluated in a general hospital setting in the Netherlands.

## Background

Approximately 15% of the Dutch population will experience a major depressive disorder (MDD) at least once in their lifetime, and 6% have experienced an MDD in the past year [1]. Murray and Lopez (Global Burden of Disease Study) stated that MDD will even become the second major cause of disability-adjusted life years in 2020 [2]. Among chronically ill patients in general hospitals, the prevalence of depression varies, ranging from 13% to 50% [3-8]. Half of all patients with chronic obstructive pulmonary disease (COPD) seem to experience clinically significant symptoms of depression and/or anxiety, and yet, comorbid MDD is frequently not identified or appropriately treated [3,5] MDD can impair the ability to seek and adhere to treatment for other medical illnesses, which can be hazardous because MDD frequently occurs in combination with a variety of other physical illnesses, including heart disease, stroke, cancer, and diabetes [9].

Strong associations have been found between physical illness and MDD. The presence of a comorbid depressive disorder has a strong influence on quality of life, self-care, adherence to medication regimens and general functioning. It raises morbidity, mortality and health care costs if combined with various chronic physical diseases [10-16]. A meta-analysis of twelve studies showed that the presence of MDD even triples the likelihood of non-adherence to medical treatment recommendations and also increases the risk of subsequent physical illness, disability, and premature death [17,18].

MDD is actually considered to be a risk factor for the onset of type 2 diabetes mellitus (DM) – depressed adults have a 37% increased risk for the development of DM [19]. Further studies have shown that higher levels of depression were associated with an increasing prevalence and severity of DM complications, such as insulin resistance, which is strongly associated with the risk of coronary heart disease [20]. MDD is one of the best predictors of hospitalization rate in older diabetic patients and among diabetic patients with a high score for depression there was even a 54% higher mortality rate than among patients with lower scores on the depression scale. However, this only applies to diabetic patients [21,22]. Among patients with heart problems the presence of depressive symptoms can be considered a risk factor for mortality and impaired cardiovascular outcome, in particular in patients with myocardial infarction [23,24].

Numerous studies have found an association between comorbid mental and physical disorders with higher resource costs [12,25-27]. Panzarino even states that MDD is one of the most costly illnesses in the USA [11]. When looking at more specific types of costs, i.e. number of visits to a physician or the length of stay in a hospital, however, the picture is less clear, and the findings are inconsistent [25].

Outpatients of a general hospital often suffer from a chronic condition. Competing demands might hamper recognition of depression in the general hospital outpatient setting in the case of chronically ill patients. Depression is still the most under-recognized and under-treated psychiatric disorder in patients with a chronic illness [28].

In 2005, the multidisciplinary guideline for depression was published in the Netherlands [29]. In this guideline, several evidence-based methods of treatment for MDD are listed, such as biological treatments, psychotherapeutic interventions, and supporting and structuring interventions. Although no specific attention was paid to the role of physical comorbidity in the guideline, the presence of physical comorbidity has been found to have no effect on the choice of treatment for MDD nor does it have an adverse effect on the outcome of treatment [30,31]

Early detection of mental or physical conditions that does lead to effective treatment increases the possibility of remission. The longer MDD remains untreated, the more difficult the treatment becomes [32]. This is even more the case with respect to serious comorbid physical illness, because physical and mental disorders can negatively influence each other's course. Nevertheless, Hyman states that approximately 90% of patients respond positively to an appropriate treatment if it is provided in the required intensity and for the right length of time [33]. Simon reviewed the social and economic burden of mood disorders, and found that MDD is associated with significant functional impairment [34]. However, he also found that effective treatment helped to restore these functions. This stresses the importance of educating physicians and nurses in the diagnosis and treatment of psychiatric disorders.

The organisation of care for comorbid physically and mentally ill patients is challenging. New concepts integrating medical and psychiatric treatment have been designed to provide care for patients with complicated medical and psychiatric disorders, so-called medical psychiatric units (MPUs) [35]. Kishi and Kathol found that in type IV type of these units, improvement in medical symptoms was comparable with improvement in general hospital wards, but that the psychiatric symptoms improved more [36].

A relatively new approach in the treatment of chronic illnesses is the so-called disease management program (DMP). A DMP can be defined as a co-ordinated unit of clinical care for chronically ill patients who are still able to actively participate in the intervention [37]. A DMP is a frequently used approach in the treatment of illnesses such as DM, arthritis, HIV/AIDS, and COPD. DMPs are also currently available for the treatment of depression [37]. Several studies have shown that a DMP for patients suffering from depression is superior compared to care as usual (CAU), in terms of reducing the severity of the depression, enhancing quality of life, maintaining employment, and adherence to medication (for at least 90 days). It has also been found to be cost-effective as well [37-39].

In the USA, a new concept, the 'collaborative care intervention' is developed [40]. Collaborative care is based on the principles of disease management and involves three main principles: firstly, active collaboration of the patient with the treatment; secondly, chained care with a built-in improvement cycle (also called 'stepped care'); and thirdly, collaboration between various medical disciplines. Katon et al. found that a collaborative care intervention, based on the 'Improving Mood-Promoting Access to Collaborative Treatment' (IMPACT) of depression for older adults with DM, was associated with more depression-free days and lower costs [41]. The IMPACT intervention consisted of a behavioural activation intervention, Problem Solving Treatment (PST) and antidepressant medication. The long-term effect of the collaborative care intervention was still associated with sustained improvement after 12 months in two-thirds of the patients [40]. The intervention further resulted in reducing the severity of the depression over time and higher satisfaction with the care provided, compared to patients receiving CAU [42]. Another study evaluating the IMPACT intervention among older adults with and without comorbid physical illnesses showed that treatment was equally effective in both groups. Hence, the presence of multiple comorbid physical illnesses had no effect on the outcome of the treatment for depression [31].

In conclusion, patients with chronic physical illnesses often experience depressive symptoms. MDD is not merely a secondary side-effect of a physical illness, but must be considered as a serious comorbid mental illness. The presence of depression has a negative effect on quality of life and increases morbidity, mortality and health care costs. However, early diagnosis and well-organized treatment of depression can have a positive influence on the costs, course, and side-effects of depression. In physically ill patients, the care has to be organized in such a way that the link with the general health care treatment can be good. Previous studies have shown that DMPs can be implemented successfully in the treatment of several – especially chronic – diseases; however, comorbid mental illnesses are not addressed in current DMP programs.

The present study will focus on patients suffering from three prevalent chronic physical diseases, namely DM, COPD, and cardiovascular diseases (CVD). Earlier studies have shown that these patients more frequently suffer from depression, compared to the general population [43-46] Treatment in a collaborative care intervention package for depression has already been realised in primary care in the USA, and has been found to be more effective than CAU. Treatment for depression will therefore be integrated in a collaborative care intervention package incorporating the principles of DMPs for the treatment of comorbid DM, CVD, and COPD. Outpatients of a general hospital who visit the DM, CVD, or COPD clinics and who have a comorbid depressive disorder will participate in this study. The collaborative care intervention package will include contracting, PST, an antidepressant algorithm, and manual-guided self-help, according to the basic model of patient and provider adherence-enhancing techniques. The interventions will be tailored to patient preferences, and compared with well-documented CAU. All treatments included in this study have individually been proven effective. The progress of the patient will be monitored and, if necessary, the treatment will be adapted. DMPs have already been proven to be effective and cost-effective as well. The goal will be to determine whether collaborative care in the general hospital is a feasible and profitable approach in terms of effectiveness and cost-effectiveness, compared to CAU.

## Methods/Design

### Objective

The primary aim of the study is to assess the effectiveness of a collaborative care model for major depressive disorders in patients with DM, CVD, and COPD in the general hospital outpatient setting. The secondary aim is to estimate the cost-effectiveness of this treatment package.

### Hypothesis

The hypothesis underlying this study is that a collaborative care model for major depressive disorder for outpatients in a general hospital setting with DM, CVD, and COPD is more effective and more cost-effective than CAU.

### Study design

The study consists of a two-armed randomised controlled trial, with randomisation at patient level. The outcome parameters will be measured by a blinded research assistant.

The patients in the intervention group will receive collaborative care from a consultative psychiatric nurse (CPN) and a psychiatrist in the department of psychiatry of the participating hospital. The control group will receive CAU from a general non-psychiatric nurse in the outpatient department. If patients in the control group are referred to a CPN in the department of psychiatry, a CPN who has not received training will treat them. Hence, trained CPNs will not influence the content of CAU, and will therefore not influence the outcome of the intervention. Since the patients and the CPNs are aware of the allocation, they can not be blinded. The patients will be assessed by means of self-report questionnaires, in order to avoid the occurrence of interviewer bias.

**Figure 1:**
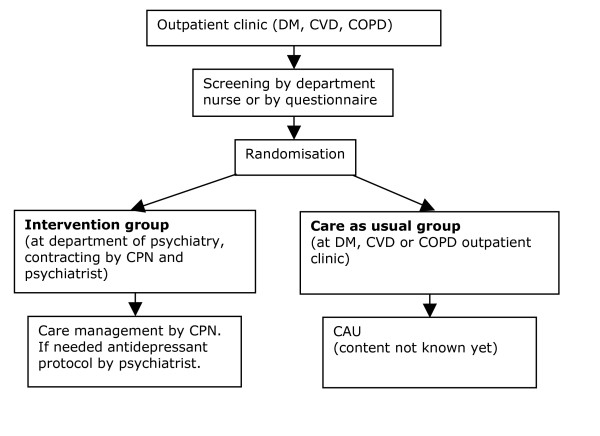
Intervention. DM: Diabetes Mellitus, CVD: Cardiovascular Diseases, COPD: Chronic Obstructive Pulmonary Disease, CPN: Consultative Psychiatric Nurse, CAU: Care As Usual

General outpatient clinic nurses will be trained in screening for depressive disorders, and will screen all patients visiting the participating departments during the inclusion period. Patients not visiting the departments during the inclusion period will be screened by mail. See Figure 1 for a flowchart.

### Recruitment of departments

The study has been set up in co-operation with the Onze Lieve Vrouwe Gasthuis (OLVG) in Amsterdam. The diabetes, cardiological, and pulmonary outpatient clinics of the OLVG will participate in the study.

A psychiatrist will be trained to provide the collaborative care intervention, together with a care manager. He will receive training in contracting (together with the care manager) and the antidepressant algorithm.

Consultative psychiatric nurses will be trained to provide the collaborative care intervention in the role of care manager. They will receive training in monitoring, care-management, PST, and contracting. The department nurses will receive training in screening for depressive disorder. The care managers in the intervention group will be closely monitored by the research team, in order to ensure that the intervention is provided correctly. There will be no care manager for patients receiving CAU.

### Recruitment of patients

All patients visiting the participating departments, who have been diagnosed with a specific chronic disease, as specified in their files, will be selected. Specific diagnoses are: DM type II in the DM department, COPD in the pulmonary department, and chronic heart failure or post-acute myocardial infarction in the cardiovascular department. Patients who meet the inclusion criteria and visit the participating departments during the inclusion period will receive an information letter, an informed consent form and the screening questionnaire (depression subscale of the 'Patient Health Questionnaire' – PHQ-9). Patients who screen positive for depression will then receive the baseline questionnaire. Patients who meet the inclusion criteria, but do not visit the participating departments will receive a package by mail which includes an information letter, an informed consent form, the screening instrument (PHQ-9), and the baseline questionnaire. In the information letter the patients are asked if they are willing to participate in a Trimbos Institute study investigating mental problems and treatment options in the general hospital setting. If they agree to participate, they will be asked to sign the informed consent form and to return it together with the completed questionnaires to the researchers.

Patients will be included in the study if they reach a cut-off score of 15 (moderate to severe depressive disorder) on the PHQ-9 [47]. For patients who reach the cut-off score, the MINI-International Neuropsychiatric Interview (MINI) will also be held by telephone to classify the symptoms [48,49] The MINI is a brief validated and structured diagnostic psychiatric interview to assess DSM-IV and ICD-10 psychiatric disorders.

In the same interview, the patients will be asked if the symptoms have been present for at least 6 weeks, can be diagnosed as major depressive disorder, and lead to general dysfunction with serious problems in at least one of the following: work, household, activities, or relationship. Therefore, the aim is to include chronically ill patients with a moderate to severe depressive disorder who dysfunction due to the depressive disorder.

**Figure 2:**
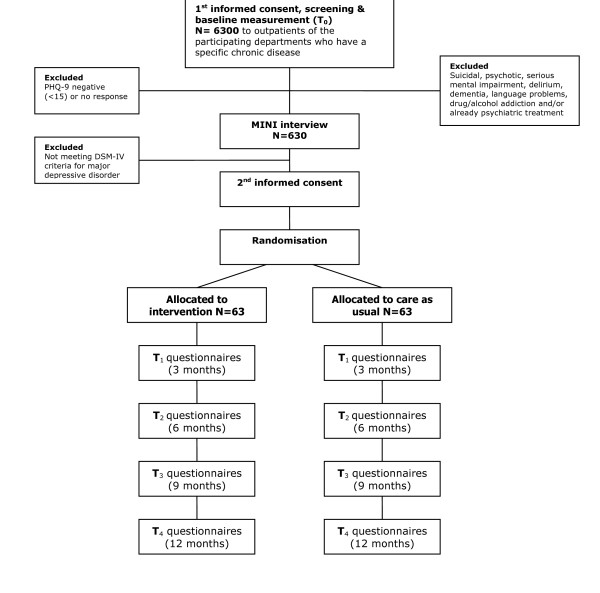
Flowchart. PHQ-9: Patient Health Questionnaire, MINI: MINI-International Neuropsychiatric Interview

For patients who are assigned to the intervention group a consultation will be arranged, first with the care manager and then with a psychiatrist, to provide more information about the treatment and to formulate a treatment plan together (contracting). Patients assigned to the CAU group will be told that they can consult their general practitioner if they feel that they need treatment, and they will be monitored (see Figure 2 for a schematic diagram). Patients will only be included in the study after they have given written informed consent. The Medical Ethical Committee of the Onze Lieve Vrouwe Gasthuis has approved the trial (study no WO06-066).

### Patient exclusion criteria

Patients will be excluded from the study if they: have insufficient knowledge of the Dutch language to fill in the questionnaires, have a serious mental impairment, are already receiving psychiatric treatment, suffer from dementia, delirium or bipolar disorder, are addicted to drugs or alcohol, are psychotic or suicidal, and/or are under 18 years of age.

### Randomisation

Patients in the participating departments who screened positive on the PHQ-9 and who have an MDD, according to the MINI, will be randomly allocated to the intervention group or the CAU group within their policlinic. Randomisation will be performed by the researcher, using a computerized method to avoid assignment bias. The patients will not be blinded for their group allocation.

### Embedding in the general hospital care setting

The collaborative care framework used in this study will last for a maximum of 22 weeks, and comprises the following four elements: a) contracting, b) antidepressant medication, c) PST, and d) manual-guided self-help. These treatments are superimposed on the basic model of patient and provider adherence-enhancing techniques.

The diabetes, cardiology, and pulmonary outpatient clinics of the OLVG will participate in this study. The elements of the collaborative care framework are divided among different disciplines in order to maximize effect of the interventions. The elements provided by each discipline performing the elements are shown in Table 1:

**Table 1:** Embedding in the hospital setting

Task	Discipline
Screening	Department nurse/Researcher
Contractor	Psychiatrist/Consultative psychiatric nurses
Care manager	Consultative psychiatric nurses
Prescription of antidepressants	Psychiatrist
Problem Solving Treatment	Consultative psychiatric nurses

### Intervention

The intervention is based on a collaborative care model [40,50-52]., which involves three main principles:

1. *Active collaboration of the patient with the treatment*. In order to investigate the motivation of the patient, the psychiatrist has to ask himself the following question: What should I do to motivate the patient to participate in this study? This principle will be addressed in the contracting phase by the psychiatrist.

2. *Chained care with a built-in improvement cycle (also called 'stepped care')*. This means that each individual step which is taken in the treatment will be evaluated. Based on the results, the next step will be undertaken, tailored to the patient's needs by the psychiatrist and the CPN.

3. *Collaboration between various medical disciplines*. The medical specialists, the department nurses, the CPNs, and the psychiatrist all work together in the diagnosis and/or treatment of the patient.

### Training

Since the treatment involves a collaborative care approach, the psychiatrist participating in the study will receive training in collaborative care, i.e. contracting and the antidepressant algorithm. The CPNs who will function as care manager will receive training in monitoring, care-management, PST, and contracting at the start of the study and the department nurses will receive training in screening for depression.

The training will be provided by members of the research group who, in turn, were trained by the IMPACT research group from Seattle, the developers of the collaborative care model used in this study [53].

### Treatment of patients in the intervention group

In the *collaborative care framework *used in this study, active collaboration of the patient with the treatment is enhanced. The care is tailored to the patient's needs within an active team, which consists of the patient, the care manager and the consulting psychiatrist. If the care manager experiences difficulties in this process, (s)he can consult the psychiatrist. Within the team the need for and assessment of the required treatment will be determined, and a treatment plan will be formulated. Subsequently, the care managers will co-ordinate the care and evaluate each step, together with the patient. According to the stepped care principle, treatment response will be monitored once every two weeks [54]. If necessary, each treatment step will be followed by a subsequent step to improve the outcome. For patients who solely receive PST, this subsequent step can involve six extra sessions of PST and/or adding antidepressant medication (AD), or switching to antidepressant medication only. The same method will be applied to patients who are treated with both PST and antidepressant medication.

The framework also includes *patient and provider adherence-improving techniques*. Provider adherence-improving interventions are administered by the research group. Provider adherence has been enhanced in an earlier study in primary care, in which psychiatrists instructed general practitioners by means of consultations, combined with phone calls and written instructions that were distributed regularly [55]. Such an approach was also found to be feasible and effective in the USA [56]. In the present study, the adherence of care managers will be improved in a similar way. Patient adherence will be improved by contracting and psycho-education [55,57].

In the present setting, the collaborative care framework includes four treatment options superimposed on adherence-enhancing techniques. The patients will have the choice of receiving antidepressant medication, but PST, contracting and manual-guided self-help are obligatory.

#### Ad a) Contracting

The care manager and the consultant psychiatrist in the hospital together inform the patient that the diagnosis is a depressive disorder. The patient will receive psycho-education about the course and the treatment options, and is offered the choice of evidence and guideline-based treatments. Depending on the patient's preferences, a joint treatment contract is formulated by the psychiatrist, the care manager and the patient. The aim of the contract is to make clear that the treatment arrangements must be adhered to by all parties involved. In an earlier study in the Netherlands, based on a collaborative care model, collaboration was indeed enhanced by contracting [55].

During the intervention phase, the patient is asked to fill in the PHQ-9 ahead of every appointment in order to evaluate the progress. The treatment steps are evaluated with the care manager. If a patient is not satisfied with the chosen treatment, (s)he can change to another form of treatment, and this will be confirmed in a new contract. If a patient wishes to withdraw from the intervention, (s)he will continue to receive CAU. All treatment choices are well documented as process measures.

#### Ad b) Antidepressant algorithm

An earlier study in the USA reported that only 46% of outpatients with care management who were treated with antidepressants at their local academic medical centre received adequate antidepressant treatment, thus reflecting a minimum standard of treatment [58]. There are further indications that chronically ill patients may require individually tailored antidepressant treatment [59]. Therefore, to enhance efficiency and adherence to the medication, a step-up antidepressant algorithm will be tailored to patients with DM, CVD, and COPD, consisting of two different types of antidepressant medication, i.e. selective serotonin reuptake inhibitors (SSRIs) and tricyclic antidepressants (TCAs).

Two antidepressants will be tested in two periods of nine weeks. If, after 18 weeks there is no improvement, the patient will be offered different kinds of treatment. In the choice of antidepressants, interactions with medication for chronic illnesses and potential palliative side-effects have been taken into consideration. If a patient is already taking antidepressants, but is still suffering from depression, the treatment is considered to be unsatisfactory, and after the required wash-out period of several weeks, the algorithm will commence. The PHQ-9 will be used to screen for and monitor the course of depression. The feasibility of the PHQ-9 to evaluate and guide a step-up algorithm has already been proven in outpatients in the primary care setting [40,60] Therefore, in the present study, the PHQ-9 will be used in the same way. The care managers will discuss the effects of the medication and the potential side-effects with the patient, and report to the psychiatrist. The psychiatrist will follow this process closely to determine whether the treatment is satisfactory.

Earlier studies have shown that the prescription of antidepressants for patients suffering from DM type I and II is safe and effective. Certain SSRIs, such as duloxetine, may also even be effective in the treatment of painful diabetic neuropathy [61,62]. However, knowledge about the interaction and side-effects of antidepressants in chronically ill patients has not yet been structured into a protocol tailored to the needs of patients with DM, CVD, and COPD.

#### Ad c) Problem Solving Treatment (PST)

Hawton and Kirkhave developed a brief psychological intervention based on the Problem Solving Therapy by D'Zurilla et al. [63] that uses the behavioural perspective, but is less time-consuming [64]. PST will be administered by nurses in 6–12 sessions, depending on the progress according to the PHQ-9 scale. The problem-solving approach consists of seven stages, and is based on the assumption that emotional problems are often induced by problems in everyday life.

Problem Solving Treatment has been found to be effective for treating MDD when provided by trained community nurses [65]. It is further significantly more effective than placebo treatment, and also more effective than antidepressant medication alone for patients in primary care [66].

The first session of PST will last for one hour, in which a clear focus of the treatment is established, based on the patient's current problems. Follow-up sessions will last for thirty minutes.

#### Ad d) Manual-guided self-help

The patient will receive a standardised treatment manual, specifically tailored to coping with depression and chronic illness, and will work through this manual. It consists of various chapters, focusing on relaxation, sleeping, exercise and cognitions, and it also gives dietary suggestions. The progress made by the patient is discussed every week or every other week between the patient and the care manager. Willemse et al. found that this kind of intervention is more effective than CAU, at least for patients in primary care with a sub-clinical depression [67]. Relaxation therapy on its own has been found to be useful as a complementary approach to depression [29].

Patients suffering from depression often experience difficulties in the sleep-wake-rhythm. The presence of these difficulties is also one of the criteria in the classification of MDD [68]. DM patients with painful peripheral neuropathy were also found to suffer considerably from sleeping problems[69]. For patients with chronic illnesses, sleeping problems are significantly correlated with poorer mental health, diminished work productivity and work quality, and greater use of the health care services [70]. Hence, the treatment of sleeping problems will be an important focus of this intervention.

Furthermore, exercise to improve general physical condition will be stimulated. Tkachuk and Martinhave extensively reviewed studies focusing on exercise therapy for patients with psychiatric disorders, and found that exercise has a positive effect, among other things, on depression [71]. In chronically ill patients with comorbid depression, exercise training was found to reduce the severity of the depression and to have a positive influence on indicators of illness risk factors [72-74]

The dietary intervention will focus on a healthy diet, losing weight and on taking omega-3 fatty acids. McElroy et al. concluded in their review that an overlap exists between mood disorders and obesity [75]. Several studies also indicate an association between omega-3 fatty acids and depression. For example, Su et al. found that omega-3 fatty acids could improve the short-term course of illness, and were well tolerated in patients with MDD [76]. The supplementation of omega-3 fatty acids was also found to be helpful in treating depression in patients with DM type 2 and in treating patients with heart diseases [77,78]

### Treatment of the patients in the control group

Half of the included patients function as a control group, in which they will receive CAU. The actual content of the CAU treatment (e.g. medication and number of contacts with physicians) will be assessed with the 'Scale assessing contacts between patients and practitioners' ('Contacten met dokters en andere behandelaars') and the 'Scale assessing medical utilization of health services' ('Vragenlijst medische consumptie') [57].

### Data collection

Data will be collected by the Trimbos Institute in co-operation with the Onze Lieve Vrouwe Gasthuis. After giving informed consent, the patients will receive assessment questionnaires by mail at baseline (T_0_), 3 (T_1_), 6 (T_2_), 9 (T_3_), and 12 months (T_4_) after inclusion. The modified 'Tailored Design Method' [79] will be used in this study to recruit the patients.

### Outcome parameters

The use of questionnaires in the hospital setting has a long tradition. Several of the questionnaires used in this study have already been used for earlier studies of patients in the hospital setting (e.g. PHQ-9 [80], IDS-SR [81], SF-36 [82]) and proved feasible. Although physical symptoms such as fatigue could be misinterpreted as depressive symptoms, in outpatients with general physical disorders no other diagnostic criteria for the diagnosis of MDD are needed [83,84].

Baseline measurements will take place before inclusion (T_0_). The first follow-up measurement will take place at the end of the treatment, i.e. after 3 months (T_1_), and further follow-up measurements will take place after 6 (T_2_), 9 (T_3_) and 12 months (T_4_).

#### 1. Primary outcome measure

##### Severity of depressive symptoms

The severity of the depressive symptoms is the primary outcome, and will be measured according to the depression sub-scale of the PHQ (PHQ-9). The PHQ-9 is a brief instrument that scores each DSM-IV criterion of a major depressive disorder on a scale ranging from zero (not at all) to three (nearly every day) [47]. The PHQ-9 has been found to be a valid and reliable instrument to measure the severity of depression in patients with different medical backgrounds [47]. In an earlier study, the PHQ-9 was used successfully for the diagnosis of MDD in diabetic patients [85].

An improvement of more than five points on the PHQ-9 can be considered a clinically relevant difference [86]. The PHQ-9 will be administered at baseline and during treatment at least every other week (before each appointment with the care manager). After treatment, the PHQ-9 will be administered every three months (3 (T_1_), 6 (T_2_), 9 (T_3_) and 12 (T_4_) months after baseline).

#### 2. Secondary outcome measure

##### Cost-effectiveness

In addition to the improvement of severity of symptoms, the cost-utility of collaborative care compared to CAU is assessed in this study. Therefore, an estimation of the direct medical costs and the costs due to production losses (productivity costs) is made. To estimate the costs the 'Trimbos/iMTA questionnaire for costs associated with psychiatric illness' (TiC-P) is used [87,88] Quality of life is assessed by the 'EuroQol' (EQ-5D) [89] and the 'Medical Outcomes Study Short Form Health Survey – 36' (SF-36) [90]. These are validated tools for measuring general health-related quality of life.

The EQ-5D descriptive system consists of five dimensions (mobility, self-care, usual activities, pain/discomfort, and anxiety/depression), each with three levels (no problems, some problems, and extreme problems), thus defining 243 (3^5^) distinct health states.

A recent study in the Netherlands measured and valuated the EQ-5D, resulting in the 'Dutch EQ-5D tariff', which is used to calculate utilities for EQ-5D health states for the cost-utility analyses of Dutch health care programmes and treatments [91,92]

The SF-36 is a self-administered questionnaire, designed for use in clinical practice and research, health policy evaluations and general population surveys [90]. It assesses eight health concepts: 1) limitations in physical activities due to health problems, 2) limitations in social activities due to physical or emotional problems, 3) limitations in usual role activities due to physical health problems, 4) bodily pain, 5) general mental health (psychological distress and well-being), 6) limitations in usual role activities due to emotional problems, 7) vitality (energy and fatigue), and 8) general health perceptions.

The cost-utility will be evaluated by relating the difference in direct medical costs per patient, receiving either collaborative care or CAU, to the difference in terms of 'Quality Adjusted Life Years' (QALY) gained, which yields a cost per QALY estimate. Furthermore, we will also estimate the cost per QALY, including the productivity costs.

#### 3. Key effect modifier

*Physical illness *is considered to be the key effect modifier, and will be measured according to the CBS list, a questionnaire developed by the Central Bureau for Statistics (CBS) in the Netherlands. The CBS list contains 28 chronic conditions, ranging from DM type II to multiple sclerosis.

#### 4. Additional outcome measures and effect modifiers

*Depressive symptoms *will also be assessed with the 'Inventory for Depressive Symptomatology-Self Report' (IDS-SR) [81]. Remission (i.e. reduction in DSM-IV criteria below the threshold for a diagnosis of depressive disorder [68]) of depressive symptoms will be measured according to the PHQ-9. Remission on the PHQ-9 is defined as a score of five or below [86].

*Somatoform presentation *will be assessed with five questionnaires. Firstly, presentation will be assessed as the number and intensity of functional somatic complaints a patient reports on the 'Bodily Complaints Questionnaire' ('Lichamelijke Klachten Vragenlijst' [LKV]) [93]. Secondly, possible comorbid somatoform disorder will be assessed with the 'Screening for Somatoform Symptoms – 7' (SOMS-7) [94]. Thirdly, hypochondria will be measured with the 'Whitley Index' (WI) [95,96]. Fourthly, health anxiety and illness behaviour will be measured with the 'Illness Attitude Scale' (IAS) [95]. Finally, pain will be measured with one scale of the SF-36 and a visual analogue scale (VAS).

*Associated symptoms of comorbid chronic illness *will be measured as fatigue, according to the Dutch version of the 'Multidimensional Fatigue Inventory' ('Multidimensionele Vermoeidheids Index' [MVI-20]) [97,98] and as disability with the 'World Health Organization-Disability Assessment Survey-II' (WHO-DAS-II) [99].

*Preference and adherence*. Preferences of the patient will be assessed according to the choices patients make in the intervention group. Patient and provider adherence will be assessed by means of a qualitative questionnaire [57]. The working relationship between the patient and the care manager will be measured with the 'Helping Alliance Questionnaire' (HAQ-II) [100], because the care manager will provide PST [101]. The attitude of the care manager towards the treatment of depressive disorder will be measured with the Depression Attitude Questionnaire (DAQ) [102,103]

*Life-events and social support *will be assessed according to the 'Social Readjustment Rating Scale' [104] to indicate the amount of change in daily life, and by two items on the SF-36 [90].

*Personality traits *will be measured according to the neuroticism and extraversion scales of the 'NEO Five-Factor Inventory' (NEO-FFI), the abbreviated version of the NEO personality inventory [105].

*Treatment in the CAU group *will be assessed with the 'Scale assessing contacts between patients and practitioners' ('Contacten met doctors en andere behandelaars') and the 'Scale assessing medical utilization of health services' ('Vragenlijst medische consumptie') [57]. Both questionnaires measure the consumption of (medical) care of the patient.

### Power calculations

In this study, the PHQ-9 will be used as the primary outcome measure. In order to detect a standardised difference of 0.5 standard deviation in the primary outcome measure (which can be considered as a clinically relevant difference), 2 × 63 patients are needed when two-sided testing and 80% power is assumed. Intention-to-treat analysis will be applied. Therefore, 126 patients will be needed. An improvement of more than five points on the PHQ-9 can be considered a clinically relevant difference [86].

### Analyses

A collaborative care model for the treatment of major depressive disorder in outpatients of a general hospital setting with DM, CVD, and COPD is more effective and more cost-effective than CAU, is the hypothesis underlying the present study. The primary outcome is the effectiveness in terms of the severity of the depressive symptoms, as measured by the PHQ-9. The secondary outcome is the cost-effectiveness, as measured with the TiC-P, the EQ-5D, and the SF-36.

#### a. Effectiveness

Continuous outcome measures will be used to measure the effectiveness of the present intervention. A t-test with intention-to-treat analysis will be performed. When missing data arises, methods like 'last observation carried forward' (LOCF) or 'multiple imputation' will be used. The central effect modifier with regard to the effect of collaborative care is comorbid chronic illness. Furthermore, somatoform presentation, associated symptoms of comorbid chronic illness, preference and adherence, life-events and social support, and personality traits will be included in the analysis as potential effect modifiers. The effect size will be estimated by Chi-square analysis and described in Cohen's d. Possible confounders, such as age, gender, immigrant status, level of education, and treatment history, will be included as variables in logistical regression analysis.

#### b. Economic evaluation

The aim of the economic evaluation is to assess the cost-effectiveness of collaborative care for the treatment of depressive disorders in the general hospital setting. A cost-utility analysis (CUA) will be applied, the results of which will be expressed as cost per QALY. The economic evaluation will be undertaken from a societal perspective, so all relevant effects and costs due to resource utilisation within the health care system (direct medical costs) and costs due to production losses (productivity costs) will be included.

Since the collaborative care intervention used in this study is new intervention, a unit price per session is not known yet. To determine a reference price, a detailed cost-price study will be performed. Therefore, we will perform measurements of time for face-to-face contacts as well as indirect time per contact (e.g. consultations of other specialists) for a total of 20 sessions. Furthermore, we will estimate overhead costs based on the information of the financial department of the hospital. This will result in an estimate of the actual costs per contact. The unit cost estimate per contact will be used as a reference price per contact for the collaborative care intervention.

The TiC-P will be used to assess the costs [87]. The TiC-P iscommonly applied in economic evaluations of treatments in mental health care. For instance, the TiC-P was recently used in a large naturalistic trial on the cost-utility of brief psychological treatment for depression and anxiety [88].

Calculating the total direct medical costs, the total number of medical contacts (outpatient visits, length of stay in hospital, use of medication, etc.) will be multiplied by unit costs of the corresponding health care services. Reference unit prices of health care services will be applied, and adjusted to the year of the study according to the consumer price index [106].

The second section of the TiC-P includes a short form of the Health and Labour questionnaire (HLQ) for collecting data on productivity losses [107]. The Short-Form HLQ (SF-HLQ) consists of three modules that measure productivity losses: absence from work, reduced efficiency at work and difficulties with job performance [108]. The number of days absent from work and the actual cost of hours missed at work due to health-related problems are valued according to the average value added per worker by age and gender per day and per hour, respectively. If respondents indicate that they have been absent for the entire recall period, data will be collected from the time when the period of long-term absence started. This additional information will be used to value the production losses according to the friction cost method [109,110]. The friction cost method takes into account the economic circumstances that limit the losses of productivity to society, which are related to the fact that a formerly unemployed person may replace a person who becomes disabled [109].

For the economic evaluation, the effects will be measured according to utility scores. In addition to the clinical outcome parameters, the utility scores will supply additional information about the impact of collaborative care on the treatment of MDD compared to the impact of CAU on the general health related quality of life. Furthermore, the results can be compared to a broad range of other health care interventions, also outside the field of mental health care.

The cost-utility will be evaluated by relating the difference in direct medical costs per patient receiving collaborative care or CAU to the difference in terms of QALYs gained, which yields a cost per QALY estimate. Furthermore, we will also estimate the cost per QALY, including the productivity costs.

In the case of missing data on costs and/or effects, and the additional uncertainty this introduces, we will use 'multiple imputation' [111]. We will use the Monte Carlo Markov Chain (MCMC) approach to impute the missing values. The uncertainty will be assessed with bootstrapping, and the results will be presented in acceptability curves [112].

### Time-frame of the study

The preparatory period will be 6 months. After approval has been received from the Medical Ethical Committee, the care managers are trained. The inclusion and intervention phase will take 22 months. The follow-up phases will be 3 (T_1_), 6 (T_2_), 9 (T_3_), and 12 months (T_4_) after inclusion. Data-analyses will take another 6 months, with interim reporting every year. The entire study period will last for 4 years.

### Ethical principles

The study has been planned, and will be carried out in accordance with the principles laid down in the Helsinki declaration (Edinburgh, Scotland amendment, October 2000).

Participation in the study is voluntarily. The patients will be informed in writing and verbally about the global features of the study, the possible benefits of participating in the experimental group, and the societal aspects of the study. The patients will be explicitly informed of the fact that they can withdraw their consent to participate at any time, without specification of any reasons, and with no negative consequences for their future treatment. Patients who wish to withdraw from the study will receive CAU.

Informed consent will be obtained twice from the patients. First, prior to completion of the baseline questionnaire, and again before inclusion in the study.

The names of the patients and other confidential information will be treated according to the medical confidentiality rules and patient data will be separated from patient names. The patients will be anonymous, because each participant will be identified in the database by a number and a code. The codes will only be available for the participating investigators.

Furthermore, all study-related data will be stored on a protected Trimbos Institute server, access to which granted exclusively to the members of the research team. The investigators will be responsible for the administration of the study. The results of the surveys will not be disclosed to the hospital staff.

The Medical Ethical Committee of the OLVG has approved of the study protocol (study no WO06-066).

## Discussion

There is a high prevalence of chronically ill patients suffering from depression, and interventions for the treatment of comorbid depression are urgently needed. Evidence for effective treatment does exist, but these are frequently not applied. The current trial is designed to implement evidence-based treatments for depression in the general hospital setting, and to test the hypothesis that these treatments are more effective and cost-effective than CAU. This is a pragmatic trial; hence, the objective of the trial is to assess the benefits of the treatment in routine clinical practice. The relationship between depressive symptoms and the course of several chronic illnesses suggests that this line of research may be of considerable importance in the field of public health.

## Competing interests

The author(s) declare that they have no competing interests.

## Authors' contributions

EKH and TBB will execute the trial and participate in the implementation. CMFC is principle investigator, conceived the study, and will assist with the implementation. LKR, HWJM, FR, and ATFB will assist with the implementation. As members of the research team, all the authors have contributed to the development of the protocol and the study design, read the manuscript, provided editorial comments and approved the final manuscript.

## Pre-publication history

The pre-publication history for this paper can be accessed here:


